# Periprosthetic bone loss: diagnostic and therapeutic approaches

**DOI:** 10.12688/f1000research.2-266.v2

**Published:** 2014-06-17

**Authors:** Loredana Cavalli, Maria Luisa Brandi

**Affiliations:** 1 Department of Surgery and Translational Medicine, University of Florence, Florence, 50139, Italy

**Keywords:** periprosthetic bone loss, osteolysis, stress shielding, subsidence, BMD, DXA, BMA, implant, arthroplasty, bisphosphonate, strontium ranelate

## Abstract

Total joint replacement surgery is being performed on an increasingly large part of the population. Clinical longevity of implants depends on their osseointegration, which is influenced by the load, the characteristics of the implant and the bone-implant interface, as well as by the quality and quantity of the surrounding bone. Aseptic loosening due to periprosthetic osteolysis is the most frequent known cause of implant failure. Wear of prosthetic materials results in the formation of numerous particles of debris that cause a complex biological response. Dual-energy X-ray Absorptiometry (DXA) is regarded as an accurate method to evaluate Bone Mineral Density (BMD) around hip or knee prostheses. Further data may be provided by a new device, the Bone Microarchitecture Analysis (BMA), which combines bone microarchitecture quantification and ultra high resolution osteo-articular imaging. Pharmacological strategies have been developed to prevent bone mass loss and to extend implant survival. Numerous trials with bisphosphonates show a protective effect on periprosthetic bone mass, up to 72 months after arthroplasty. Strontium ranelate has been demonstrated to increase the osseointegration of titanium implants in treated animals with improvement of bone microarchitecture and bone biomaterial properties.

## Introduction

Endosseous implantation is one of the most common procedures in orthopedics and dentistry. The ever expanding aging population has also led to an increasing need for total joint replacements ^1^. Unfortunately, the introduction of a prosthesis or a dental implant inevitably alters the physiological transmission of loads to the surrounding bone, which starts a remodeling process, resulting in reduction in bone mineral density (BMD) ^2^.

Aseptic loosening due to bone destruction around the prosthesis has been established as the main cause of implant failure ^3– 6^. Mechanical, thermal and chemical intraoperative damage induces necrotic phenomena on the periprosthetic bone, which takes approximately 3 months to repair ^2, 7^. Then the osteocytes, acting as mechanoreceptors, translate the mechanical stimulus into an electrical signal, either activating osteoclasts (OCs) in bone areas no longer subjected to physiological loading or stimulating osteoblastic cell lines where bone is stressed, with consequent hypertrophy ^2, 8– 10^.

Despite being a widespread practice, joint arthroplasty almost unavoidably involves a loss of surrounding bone, which can cause periprosthetic fractures resulting in reduced function, subsequent morbidity and increased risk of mortality ^11^. After Total Hip Arthroplasty (THA), for example, periprosthetic fractures occur in 0.8% of patients at 5 years, and 3.5% at 10 years ^11, 12^. This is the third most common reason for re-operation ^13, 14^, while implant failure by aseptic loosening could be expected in 3–10% of cases within 15 years ^11, 12^.

A better understanding of the biological basis of peri-implant osteolysis has allowed the development of therapeutic strategies to prevent periprosthetic bone loss, in particular with Bisphosphonates (BPs) and Strontium Ranelate (SrRan).

This paper will cover the biological basis of periprosthetic bone resorption, diagnostic techniques and preventive or therapeutic approaches, both from a pharmacological and surgical point of view.

## Pathogenic mechanisms underlying aseptic loosening of implants

Risk factors for periprosthetic bone destruction include osteoporosis, rheumatoid arthritis, revision surgery and stress shielding. These lead to a resorption process in bone areas that are no longer mechanically subjected ^15^. The basis of this process includes mechanical and biological factors ^16^.

Several reports have shown that the cellular responses to biomaterial wear particles play an important role ^16^. Particles ranging from 0.2 to 10 μm in diameter undergo phagocytosis by macrophages ^17^.

*In vitro* studies of macrophage cultures clearly indicate that smaller particles of polymethylmethacrylate (PMMA) and polyethylene, materials used in implants, (< 20 μm) elicit a significantly increased inflammatory cytokine response, as indicated by increased release of Tumor Necrosis Factor (TNF), IL-1, IL-6, prostaglandin (PG)E2, matrix metalloproteinases, receptor activator of nuclear factor kappa-B ligand (RANKL) and other factors that affect osteoclast differentiation and activity ^16, 18– 21^. Moreover, direct biologic interaction between particles and the cell surface is sufficient to activate osteoclastogenic signaling pathways ^16, 18, 22^, causing bone resorption and periprosthetic BMD loss.

Bone turnover markers, due to their high intra-individual variability, have a limited predictive value concerning the extent of periprosthetic bone loss ^23^. However, the immediate high postoperative activity of osteoclasts is confirmed by a study on 53 patients followed for 12 months after THA with a cemented femoral stem ^23^. The study showed an early postoperative increase of C-terminal telopeptides of type I collagen (CTX-I) (markers of bone resorption), where the highest level was noted (+21%) 3 weeks after THA, then decreased at 8 weeks after THA (-7% from preoperative levels). This increase was significantly correlated with the bone loss measured by DXA in the calcar region ^23^. These data suggest that a postoperative antiresorptive treatment administered for the period of increased CTX-I levels could prevent periprosthetic bone loss ^23^.

Stress shielding is also considered as a potent stimulator of bone resorption. After a total hip arthroplasty, the stem geometry of the implant plays a key role in the load transfer to the femur and consequently in femoral remodeling ^24, 25^.

Conflicting results have emerged regarding the hypothesis that the amount of periprosthetic bone remodeling can be influenced by different factors, including sex, age, weight, Body Mass Index (BMI) and bone mass ^26– 30^. The data on the role of stem design are more consistent ^24^.

## Imaging techniques for the evaluation of periprosthetic BMD loss

### DXA

**Figure 1.**
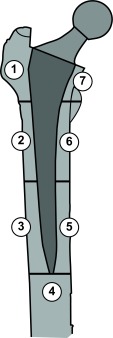
The seven Gruen zones: a model for the evaluation of hip periprosthetic bone remodeling.

Dual-energy X-ray Absorptiometry (DXA) is considered the most reliable tool to evaluate bone remodeling after THA using implants with different stem designs ^31, 32^. It is also used to assess the effectiveness of these treatments by comparing the medium-term bone density changes between treated and untreated groups. A special piece of software named “metal-removal” enables DXA to analyze periprosthetic bone using seven conventional Regions Of Interest (ROI) called Gruen zones ( Figure 1) ^33, 34^.

Thanks to improvements in software and technology, bone densitometry examinations by DXA may actually allow the detection of periprosthetic bone remodeling that cannot be observed in conventional radiographs as DXA provides an accurate measurement of total and regional periprosthetic BMD after THA ^24, 35– 38^.

DXA scanning is usually performed with the patient in the supine position, the leg placed in a standardized support to ensure a neutral position ^38^. Analysis of the 7 periprosthetic Gruen zones is the most commonly used protocol to evaluate bone remodeling after the implantation of conventional femoral stems ^24, 31, 40, 41^. As shown in Figure 1, in the horizontal plane, the tip of the lesser trochanter defines the distal border of zones 1 and 7. The midpoint between the lesser trochanter and the tip of the stem defines the border between zone 2 and zones 3, 5 and 6. Zone 4 represents the total bone area 20 mm distally from the tip of the stem. Vertically, the center axis of the femur divides the medial and lateral zones ^39^.

Postoperative measurements are commonly used as baseline values and the measurements at follow-up are expressed as a percentage of the baseline measurements ^39, 42^. However, in cross-sectional studies, the controlateral unoperated hip has also been analyzed to obtain individual comparative BMD values ^24, 40, 43, 44^.

Although DXA is regarded as the most accurate method for the detection of small alterations in bone mineral density around hip prostheses ^39^, its metal-removal software also provides peri-prosthetic measurements for knee arthroplasty ^30, 45– 49^. No DXA protocol is available for ankles, shoulders, elbows, or wrists.

### X-ray CT imaging with metal subtraction

The study of periprosthetic bone by X-ray techniques can be hindered by artefacts due to the presence of inorganic material in the scan plane. Metal artefacts can be reduced by the use of suitable filters acting during the acquisition, which allow reducing the radiation spectrum and the dynamic range from the beam itself. There are also algorithms to estimate the error due to the presence of artifacts in the acquired images. A method of suppression of high-density artefacts not requiring the introduction of extra bone artefacts was described by Wey and colleagues ^47^. Bone pixels are isolated and segmented by thresholding, then artificial numbers are assigned to them, while the projection profile of metal object is removed ^47^.

The effects of femoral adaptive bone remodeling after THA have also been assessed by quantitative computer-tomography assisted osteodensitometry, a method able to differentiate cortical and cancellous bone structures ^48^. The CT scans were downloaded onto a dedicated software (CAPPA postOP, CAS Innovations AG, Erlangen, Germany), and automatically evaluated on the basis of an adaptive tracer algorithm that outlined the contour of the outer and inner cortical bone as well as the prostheses, completed by manual correction of metal artefacts ^48^.

Moreover, an artifact reduction algorithm was developed in ^18^F-FDG studies by PET/CT scanners ^49^. This method, which allows the anatomical information from CT to be combined with metabolic PET data, is particularly useful in cancer staging, where the improvement in detectability of small lesions located near metal hip implants by this algorithm is very important ^49^.

### Bone Microarchitecture Analysis (BMA)

An innovative device has recently been developed, the BMA, which combines ultrahigh resolution 2D digital X-Ray images and a set of trabecular bone texture analysis parameters, such as 2D fractal analysis (H mean), Co-Occurrence (COOC) and Run Length Encoding (RLE), thus providing a bone microarchitecture quantification independent of bone density measurement ^50^.

With a resolution near 100 µm, BMA visualizes the bone structure at the trabecular level, allowing the *in vivo* micro-analysis of human bone structure and abnormalities, such as fracture lines that are often uncertain or ignored. All the joints can be examined (i.e. spine, hip, knee, ankle, shoulder, elbow, wrist and phalanges). The micrometric accuracy in the visualization of joint interspace reflects cartilage thickness, useful for the diagnosis and follow-up of osteoarthritis (OA). The digital X-Ray detector allows examination at a very low dose (effective dose < 2 μSV for a heel exam) due to its excellent Detection Quantum Efficiency (DQE). This performance, added to the quality of a high frequency X-Ray generator, is obtained in less than 1 second exposure time, while the image processing is achieved in less than 2 minutes, facilitating patient workflow and improving productivity ^50^.

Although not currently widespread, except in few research centers specialized in bone diseases, the employment of BMA may represent a promising device for the study of periprosthetic bone analysis of any joint as well as for arthritis, osteoarthritis and altered bone healing.

## Therapeutic strategies for enhancing bone mass recovery after arthroplasty

### Surgical approaches

Since the stem geometry of the implant is believed to play an important role in load transfer to the femur, biomechanical tests ^51– 53^ and radiographic studies ^54^, followed by DXA analyses, have been conducted on patients subjected to THA. These studies suggest that the ultra-short implant (which has a more anatomical proximal fit without having a diaphyseal stem with distal cortical contact) can provide immediate postoperative stability and a more physiological load distribution, thus increasing periprosthetic BMD in the medial regions over time ^24^, preserving bone mass and stimulating trabecular bone apposition ^24, 53, 55^. The presence of the lateral flare makes the diaphyseal stem with distal cortical contact almost unnecessary, thus increasing periprosthetic BMD in the medial regions over time ^24^.

Albanese and colleagues ^24^ assessed bone remodeling in patients subjected to two metaphyseal implants, type 1 with a very short stem and type 2 with no diaphyseal stem. Using a 5-ROI protocol of DXA analysis, they found that ultra-short implants can provide immediate postoperative stability and a more natural physiological load distribution in comparison with conventional anatomic implants, thus increasing periprosthetic BMD in the medial regions over time.

After stem design, the most important factor known to influence periprosthetic BMD is the fixation of the implant ^27^. A fundamental feature that enables fixation is the porous surface of the prosthesis ^56^. Cementless THA is increasingly popular. The high rate of osteolysis, aseptic loosening and revision associated with earlier uncemented femoral components has been greatly reduced by using better designed implants incorporating circumferential porous coating ^56^. Moreover, proximal femoral fixation has been shown to prevent stress shielding and a tapered distal tip reduces thigh pain ^56^. The mid-term outcome of a modular, cementless, proximally hydroxyapatite-coated, anatomic femoral stem in THA was reported by Cossetto and Goudar (Modulaire Biconique Anatomique, MBA Groupe Lépine, France) ^56^. They showed that the modularity of the neck of this femoral component is helpful in both primary and revision settings. In primary procedures, after implantation of the stem, correction can still be made in leg length and offset. In revision procedures, the modularity of the neck facilitates adjustments in leg length, offset and neck version without the need to extract a well fixed femoral component. It also facilitates access to the acetabular component by way of removal of the modular neck and head ^56^. In that study, in case of dislocation, changing the modular neck and head avoided more extensive revision requiring removal of a well-fixed femoral stem. Patients were evaluated pre- and post-operatively (at 6 weeks, 3 months, one year, 2 years, 5 years and 10 years), with a clinical evaluation (pain, range of movement, and ability to walk) based on Merle d’Aubigne and Postel scores ^57^ and by anteroposterior and lateral weight-bearing radiographs, in which the femoral component was analyzed according to the 7 zones of Gruen. Contrary to the increased rate of revision in modular hip systems found in the Australian Orthopaedic Association National Joint Replacement Registry, the modular, cementless, proximally hydroxyapatite-coated, anatomic femoral stem provided predictably stable fixation with excellent mid-term outcome ^58^.

Similarly, Lerch and colleagues ^33^ conducted a prospective densitometric study by DXA in a group of patients who underwent unilateral bicontact stem implantation. This is a cementless implant made of a titanium forged alloy (Ti6A14V), with a proximal microporous pure titanium plasmapore coating. Despite small signs of stress shielding observed at the tip of the stem, it has shown to provide adequate proximal bone stock preservation ^33^.

Studying knee arthroplasty is rather difficult when compared to studying THA, due to the position of the patient required for the exam. Full extension of the knee is not possible for most patients in the first days after surgery, therefore while deficits in extension normalize with rehabilitation, individual knee flexion between the baseline and follow-up investigations may be different ^47^. A clinically applicable soft foam positioner designed to ensure rotational stability and allow for slight flexion (i.e. 25°) may be safe for clinical use, because this position can be obtained with all normal total knee arthroplasty (TKA) patients both in the early period after surgery and in later follow-ups ^47^.

However, a prospective cohort study conducted by Windisch and colleagues ^48^ described the changes in bone density over the course of time following a cement-free TKA based on a functional categorization of the measurements in terms of defined ROI by means of DXA. The seven regions were defined as indicated in Table 1.

**Table 1.** Seven Regions of Interest (ROI) for bone mineral density measurements after total knee arthroplasty ^48^.

ROI 1	Distal femoral region above the prosthesis
ROI 2	Lateral proximal region below the tibial prosthesis tray
ROI 3	Lateral distal region below the tibial prosthesis tray
ROI 4	Medial proximal region below the tibial prosthesis tray
ROI 5	Medial distal region below the tibial prosthesis tray
ROI 6	Zone adjacent to the prosthesis below the tibial stem
ROI 7	Distal tibial region below the prosthesis

A further aim of that study was to examine the associations between the defined parameters of age, sex, severity of arthrosis, and axis alignment. At 12 months after surgery, a high severity of osteoporosis was associated with low absolute values for periprosthetic bone density. Women demonstrated a lower absolute periprosthetic BMD value than men. The preoperatively determined femur and tibial average Cortical bone Marrow Index (CMI), the varus angle and the BMI showed no significant correlation with the absolute or relative changes of periprosthetic bone density ^48^. Statistical analysis revealed that the most significant changes occurred within the first 3 months postoperative with the highest bone density loss found in the region of the proximal medial tibia ^48^.

Another group ^49^ has studied the effects of Unicompartmental Knee Arthroplasty (UKA), which has received renewed interest for medial OA within the last decade. UKA has been traditionally used in older, non-obese patients with a sedentary lifestyle. UKA is advantageous as only the severely damaged compartment is replaced and the bone stock is preserved, which is associated with fast recovery times ^49^. Moreover, improvements in surgical technique, implant materials and prosthetic design have made UKAs more durable and reliable ^49^.

The group measured BMD using DXA and data were collected from multiple ROIs for each patient at several intervals during the first 7 postoperative days. The highest femoral periprosthetic bone loss rate was observed during the first 3 months after UKA. However, BMD changes from 2–7 years were not significant. In particular, there was a significant loss of BMD from distal femoral sites after UKA, while BMD changes were minor in the tibial metaphyseal regions, consistent with a mechanical axis balance between the medial and lateral sides of the tibia. Further, porous tantalum tibial components maintained better periprosthetic BMD compared with cemented tibial implants ^49, 59^.

### Pharmacological strategies to prevent aseptic loosening

In combination with improvements in implant integration, strategies to target the cellular components (osteoblasts and osteoclasts) that contribute to implant failure should be implemented ^60^. In this regard, it should be noted that differentiation of bone marrow macrophages into mature osteoclasts requires recognition and binding of osteoblasts, fibroblasts, and T cell secreted factor RANKL by its cognate receptor RANK, which is expressed on the surface of osteoclast precursors ^61– 63^. Another osteoblastic factor, namely osteoprotegerin (OPG), acts as a decoy receptor by binding to RANKL and reducing its bioavailability. On the other hand, binding of RANKL to RANK stimulates induction of several intracellular pathways by this receptor, leading to activation of key transcription factors, most notably NF-κB ^22^.

It is known that NF-κB activation, when induced by factors such as TNF and PMMA particles, exacerbates osteoclastogenesis and inflammatory responses ^22^. In this context, a review by the group of Abu-Amer considers three kinds of approaches ^22^. The first involves targeting OC precursor cells, which are brought to inflammatory sites by circulating cytokines. The second entails targeting precursors that are stimulated by the particle-mediated cellular response to differentiate into OCs. The third approach involves targeting activation mechanisms of mature osteoclasts ^22^.

An example of the first strategy is the application of RANKL decoy molecules such as OPG and the soluble fusion protein RANK-Fc ^64, 65^, but the successful preclinical findings, i.e. the ability to prevent and reverse wear debris-induced osteolysis, have not been confirmed by clinical trials. At present, the monoclonal antibody anti-RANKL is available, known as ‘denosumab’, which shows significant effectiveness in the inhibition of bone resorption due to osteoporosis ^66^. Transduction of a dominant-negative form of the NF-κB inhibitory protein, IκB, by retaining NF-κB in the cytoplasm, has been revealed to be able to block osteoclast formation and activity ^16, 67– 69^. Another viable approach is to block activation of the upstream IKK complex, which is responsible for phosphorylation of IκB and subsequent activation of NF-κB, by introducing a small peptide that hinders assembly of the IKK complex ^16, 70^. Notably, administration of the dominant negative IκB protein or the IKK inhibitory small peptide to arthritic mice blocks bone erosion and particle-induced osteolysis of calvaria in mice ^16, 71^.

Further targets of therapy are NF-κB mediated genes. Recent studies have revealed that proinflammatory cytokines such as TNF act directly on some of these genes and their products, in particular c-src and NF-κB, to accelerate osteoclast formation and cause a strong osteoclastic response ^71^. Selective inhibitors of the c-src tyrosine kinase have shown great promise in reducing osteoclast activity ^16, 72, 73^.

Another promising approach involves the use of bisphosphonates (BP) ^2, 16, 74^, potent anti-resorptive drugs widely used in the treatment of osteoporosis, which inhibit osteoclast function and induce their apoptosis. In animal models subjected to implantation ^75– 77^, oral BP showed reduced radiographic periprosthetic radiolucency, as inhibiting debris-induced osteolysis, although the levels of PGE2 and IL-1 remained elevated in tissue cultures from these implants. These studies have served as a basis for clinical trials using alendronate, one of the most commonly used BPs, in patients with radiographically evident osteolytic lesions. In other studies, bone loss around implants was prevented and treated by alendronate ^78– 81^.

More recently, human clinical trials have revealed the efficacy of BPs in reducing particle-induced osteolysis over the first year of life of cemented and cementless hip and knee replacement prostheses, with better and more durable results when treatment was started early after surgery and continued for over 6 months ^2, 82, 83^. Moreover, *in vivo* trials showed a direct action of some BPs in stimulating the osteoblastic proliferation, which might play an essential role in increasing periprosthetic bone ingrowth ^84^. The mechanism by which BPs are supposed to act on the osteoblasts is by up-regulating the expression of genes coding the synthesis of some morphogenetic proteins, including BMP-2 ^85^.

Muratore and colleagues assessed the effect of ibandronate, another type of BP that can peculiarly be administered either orally or intravenously, with extended dosing intervals, thanks to its high affinity for the bone mineral component and its consequent long-term persistence in the skeletal tissue, which therefore ensures excellent adherence to therapy ^2^. Thirty-five women over 60 years old, not necessarily suffering from osteoporosis, were subjected to THA. They were examined by DXA at the 15 ^th^ day after surgery (T0) and at 6 and 12 months, either at the spine, contralateral femur or periprosthetic femur, both totally and at the 7 Gruen regions. Of these patients, 19 patients received 3 mg ibandronate intravenously within 5 days after surgery and then passed to oral administration with a monthly dose of 150 mg, plus calcium carbonate (1 g) and cholecalciferol (880 IU) supplementation. The other 16 patients formed the control group and were treated only with calcium carbonate (1 g) and cholecalciferol (880 IU) supplementation. As a result, a reduction in the BMD was observed over the first 6 months from T0 in both groups; smaller reductions were observed in the treated group (-7.7% compared to the control group). In contrast, at 12 months, a marked trend reversal was observed, with a statistically significant BMD percentage recovery compared to the baseline value at T0 of about 1.74% of the global BMD in the treatment group. This was more evident in region R1 (+3.81%) and in the lateral metaphyseal region (R2) (+4.12%). On the other hand, no global BMD recovery was observed in the control group, which had virtually stabilized compared to values at 6 months ^2^.

Considering that periprosthetic remodeling occurs within the first 6–12 months after surgery, this study therefore concluded that ibandronate reduces periprosthetic resorption, in particular in the medial metaphyseal region (calcar and lesser trochanter), the one at greater risk with respect to the life of the prosthesis.

Bisphosphonates, which are chemically stable analogues of inorganic pyrophosphate, can be classified into at least two groups with different molecular modes of action. The simpler non-nitrogen-containing bisphosphonates (such as etidronate and clodronate, BPs of first generation) can be metabolically incorporated into nonhydrolysable analogues of adenosine triphosphate, which interfere with adenosine triphosphate-dependent intracellular pathways ^86^. The more potent nitrogen-containing bisphosphonates (including pamidronate, alendronate, risedronate, ibandronate, and zoledronate) are not metabolized in this way but inhibit key enzymes of the mevalonate/cholesterol biosynthetic pathway, such as farnesyl pyrophosphate synthase, compromising the function of essential intracellular messengers, thus causing osteoclast inactivation and apoptosis ^87^.

A meta-analysis ^11^ of 14 randomized controlled trials employing BPs after joint arthroplasty found that the protective effect of these drugs, probably modified by BP generation and the prosthesis location, could persist in a middle-term follow-up after surgery and for 18 to 70 months after drug discontinuation. The efficacy was more potent for amino-BPs, than for the first generation of BPs ^11^.

However, since the trials did not address the clinically relevant outcomes, it is imperative to perform a randomized clinical trial with an adequate number of patients and sound methodology in order to establish the definitive role of BPs in joint arthroplasty, and make recommendations for their optimal administration. It is also necessary to better understand the mechanisms of their actions and potential side effects ^11^.

Moreover, prolonged use of BPs has recently been associated with severe suppression of bone turnover ^88– 90^, alterations in normal collagen cross-linking and matrix heterogeneity ^91– 93^, reduced vascularity ^94, 95^ and decreased cortical bone toughness ^95– 98^, as well as a small number of subtrochanteric or diaphyseal femoral fractures ^95, 99– 102^. The exact mechanism of these ‘atypical’ fractures is unknown. One theory is that they occur in the subtrochanteric region of the femur because it is subject to high bending forces ^103– 106^. The latter would cause the formation of micro-cracks, normally repaired through bone remodeling. Bisphosphonates’ suppression of bone turnover results in a failure to repair these micro-cracks ^102^.

Calcitonin is a 32-amino acid polypeptide hormone (produced predominantly in C-cells of the thyroid gland) which mainly acts by inhibiting osteoclast function ^107^. It has been shown that 200 IU of salmon calcitonin administered nasally decreases osteoporotic fractures ^108, 109^. A Finnish clinical trial ^107^ randomized 60 patients who underwent THA using cemented Exeter prostheses into a treatment group (200 IU salmon calcitonin + calcium 500 mg) and placebo group (inactive nasal spray + calcium 500 mg) for 6 months. They were followed with DXA, bone turnover markers and dynamic histomorphometry on bone biopsies taken from the femoral neck at the time of discharge, after 6 and 12 months. Calcitonin was not shown to promote any additional value on calcium substitution in preventing aseptic osteolysis ^107^.

Another antiosteoporotic treatment, Strontium Ranelate (SrRan), was shown to be of considerable interest in investigations to improve implant osseointegration ^1^. The beneficial effects of SrRan have previously been reported in various animal models, where it has been shown to prevent bone loss by maintaining bone formation at a high level and inhibiting bone resorption ^1, 110– 114^. These *in vivo* results were consistent with *in vitro* data which show that SrRan reduces bone resorption by osteoclasts and increases bone formation by osteoblasts ^115– 118^. In particular, SrRan has been shown to enhance preosteoblastic cell replication and osteoblastic differentiation and to decrease abilities of osteoblasts to induce osteoclastogenesis, both through the calcium-sensing receptor and an increase in the OPG/RANKL ratio ^116– 118^.

It has also been demonstrated that SrRan is able to improve bone biomechanical and structural properties ^119^. Furthermore, treatment with SrRan is not associated either with osteonecrosis of the jaw or with low energy atypical fractures of the femur ^1^.

A group from the Bone Division of Geneva ^1^ showed that SrRan significantly improves mechanical fixation of titanium implants inserted into the tibias of female rats, with both a positive effect on bone microarchitecture and on bone biomaterial properties in the vicinity of the implant. SrRan increased pull-out strength compared to controls (+34%), with a significant improvement of bone microarchitecture around the implant, a more plate-shape structure and an increase in bone-to-implant contact (+19%) ^1^.

Another study ^120^ was designed to evaluate the effect of systemic SrRan treatment on fixation of hydroxyapatite (HA)-coated titanium screws in ovariectomized (OVX) rats. The OVX rats were randomly divided into the following groups: OVX (without treatment), OVX+SRL (treated with a low SR (i.e. SrRan) dose of 500 mg/kg/day) and OVX+SRH (with a high SR dose of 1000 mg/kg/day). Micro-CT and biomechanical push-out tests were performed twelve weeks after treatment, in order to evaluate bone blocks with implants. The two groups treated with SR showed an increase of bone volume ratio, osseointegration and maximal force, compared to OVX animals, suggesting that SrRan treatment can improve HA-coated screw fixation dose-dependently in OVX rats and facilitate the stability of the implant in the osteoporotic bone ^120^.

These results may support the potential benefits of SrRan in enhancing osseointegration in orthopaedic and dental surgery.

## Conclusions

Aseptic loosening, due to periprosthetic osteolysis, is the most common cause of implant failure.

Among the other clinical and biomechanical criteria, bone status should be considered before proceeding with arthroplasty, in order to select the most adequate implant model as well as to evaluate the necessity of an anti-osteoporosis therapy.

Dual-energy X-ray Absorptiometry allows evaluation of bone density around hip or knee prosthesis, and further data may be provided by the new Bone Microarchitecture Analysis device.

The comprehension of the principal mechanisms of periprosthetic bone loss has led to the development of pharmacologic strategies aiming at the enhancement of bone mass recovery after surgery and consequently to the prolongation of implant survival.

BPs, potent anti-resorptive drugs widely used in the treatment of osteoporosis and other disorders of bone metabolism, were shown to be particularly effective in reducing periprosthetic bone resorption in the first year after hip and knee arthroplasty, both cemented and cementless.

SrRan, due to its antiresorbing and bone-forming activity, also promises to facilitate the stability of dental and joint implants in both healthy and osteoporotic bone.
