# Supplementary material for: Periprosthetic bone loss: diagnostic and therapeutic approaches
Source: F1000Res. 2014 Jun 17;2:266. [Version 1] doi: 10.12688/f1000research.2-266.v2 (PMC4304431; doi:10.12688/f1000research.2-266.v2)
Supplement: Peer review history

# Peer review: Referee response for version 2

**DOI:** 10.5256/f1000research.4823.r7008

**Published:** 2015-01-15

**Authors:** P Edward Purdue

**Affiliations:** 1 Division of Research, Hospital for Special Surgery, New York, NY, USA

This is a nice review that more addresses bone loss directly following TJR as opposed to the consequences of wear driven osteolysis that can initiate many years after surgery. Nevertheless, the discussions on treatment approaches are useful. Adding that denosumab is now entering clinical trials for peri-implant osteolysis (clinicaltrials.gov) might be warranted.

I have read this submission. I believe that I have an appropriate level of expertise to confirm that it is of an acceptable scientific standard.

# Peer review: Referee response for version 2

**DOI:** 10.5256/f1000research.4823.r5158

**Published:** 2014-08-13

**Authors:** Jason Wong

**Affiliations:** 1 Institute of Inflammation and Repair, University of Manchester, Manchester, UK

This review nicely covers the current work in enhancing bone integration with modern implants, however this field will be truly exciting over the next 5-10 years as we will see a vast improvement in device design with biologic materials with rapid prototyping and tissue engineering.

I have read this submission. I believe that I have an appropriate level of expertise to confirm that it is of an acceptable scientific standard.

# Peer review: Referee response for version 1

**DOI:** 10.5256/f1000research.1270.r4886

**Published:** 2014-05-27

**Authors:** Javad Parvizi

**Affiliations:** 1 Department of Orthopaedics, Rothman Institute, Thomas Jefferson University Hospital, Philadelphia, PA, USA

This is a comprehensive evaluation of periprosthetic bone loss in mostly total hip arthroplasty that is helpful for orthopaedic surgeons. Currently, most orthopaedic surgeons only evaluate bone loss with plain radiographs. Having the knowledge to use DXA and BMA may be helpful in selective cases, although ordering advanced imaging on all patients may not be clinically necessary. Additionally, being able to combat bone loss with therapeutic treatments such as bisphosphonates and strontium ranelate may prove useful in a select patient population for a specified duration of time. Overall, this is a helpful article with cutting edge technology that may be useful for imaging techniques to better evaluate bone loss, and holds potential for evaluating future treatments to prevent and restore bone loss.

**Title/Abstract**: Both are appropriate

**Imaging techniques for the evaluation of periprosthetic BMD loss**:

- This should include a section on x-ray imaging and CT imaging with metal subtraction.

- What is the accessibility of BMA? What type of facilities have this? Are there studies comparing this technology with other imaging techniques with implants?

**Therapeutic strategies for enhancing bone mass recovery after arthroplasty**:

- Good sections on surgical and pharmacological considerations

- Under “ *Pharmacological strategies to prevent aseptic loosening*,” the authors mentioned the use of OPG and the soluble fusion protein RANK-Fc. However, there are no studies presented evaluating this in the setting of implants and only in the osteoporosis model. This may not be an appropriate to mention in this article (in comparison to the dominant negative IκB protein or the IKK inhibitory small peptide, which has been demonstrated to block particle-induced osteolysis).

- It would be helpful to provide the mechanism of action of Strontium Ranelate.

**Conclusions**: There should be a sentence on imaging, as mentioned in the article.

I have read this submission. I believe that I have an appropriate level of expertise to confirm that it is of an acceptable scientific standard, however I have significant reservations, as outlined above.

# Response

**Published:** 2014-05-27

**Authors:** Loredana Cavalli

**Affiliations:** Dep. of Surgery and Translational Medicine, University of Florence, Italy, Italy

The authors would like to thank the referee for his response.

- As regards BMA technique, it is not currently widespread for a clinical use, except in few research centers specialized in bone diseases, like ours at Orthopedic Trauma Center in Florence, Italy. As its entrance is very recent, and its potential applications are very wide, it is not yet available a protocol to compare this technique with other ones for peri-prosthetic bone study. So, at the end of the paragraph *Imaging techniques for the evaluation etc., *before the last sentence *, *we would add: " *Although not currently widespread, except in few research centers specialized in bone diseases*, the employment of BMA..."
- About metal subtraction option in X-ray and CT imaging, as it represents a real possibility to correct artifacts, we are preparing a brief paragraph to add: thank you for the suggestion!
- OPG and RANK-Fc were the first molecular strategy, so we would cite them, adding the sentence " *but the successful preclinical findings, i.e. the ability to prevent and reverse wear debris-induced osteolysis, have not been confirmed by clinical trials*".
- Regarding Strontium Ranelate, we would add: " *In particular, SrRan has been shown to enhance preosteoblastic cell replication and osteoblastic differentiation and to decrease abilities of osteo­blasts to induce osteoclastogenesis, both through  the calcium-sensing receptor and an increase in the OPG/RANKL ratio *(new ref.: Brennan TC, Rybchyn MS, Green W, Atwa S, Conigrave AD, Mason RS. Osteoblasts play key roles in the mechanisms of action of strontium ranelate. *Br J Pharmacol*. 2009;157(7):1291–1300)."
- In the conclusions, the sentence " *Dual-energy X-ray Absorptiometry allow to evaluate bone density around hip or knee prosthesis, and further data may be provided by the new device called Bone Microarchitecture Analysis*." can be added after the second paragraph.

We will provide a new version of the paper with all these corrections as soon as possible.

# Peer review: Referee response for version 1

**DOI:** 10.5256/f1000research.1270.r2668

**Published:** 2014-01-20

**Authors:** Jason Wong

**Affiliations:** 1 Institute of Inflammation and Repair, University of Manchester, Manchester, UK

I would like to congratulate Loredana Cavalli and Maria Luisa Brandi on this really quite enjoyable review. For me, I found it quite comprehensive but there were some minor omissions such as the role of electrical charge on bone integration and role of tissue engineering and rapid prototyping on prosthetic design in the future. I would also have liked to have more information from the lessons learnt from failed implants in terms of the pathobiology at the interface. Otherwise it was a good review of the literature and worthy of publication.

I have read this submission. I believe that I have an appropriate level of expertise to confirm that it is of an acceptable scientific standard.

# Response

**Published:** 2014-05-27

**Authors:** Loredana Cavalli

**Affiliations:** Dep. of Surgery and Translational Medicine, University of Florence, Italy, Italy

The authors would like to thank the referee for his response.
